# Bone Regeneration in Defects Created on Rat Calvaria Grafted with Porcine Xenograft and Synthetic Hydroxyapatite Reinforced with Titanium Particles—A Microscopic and Histological Study

**DOI:** 10.3390/jfb16040146

**Published:** 2025-04-19

**Authors:** Antonia Samia Khaddour, Emma Cristina Drăghici, Mihaela Ionescu, Cristina Elena Andrei, Răzvan Eugen Ghiţă, Răzvan Mercuţ, Oana Gîngu, Gabriela Sima, Lavinia Toma Tumbar, Sanda Mihaela Popescu

**Affiliations:** 1 Department of Oral Rehabilitation, University of Medicine and Pharmacy of Craiova, 200349 Craiova, Romania; antoniasamia11@gmail.com (A.S.K.); razvan.ghizza@yahoo.com (R.E.G.); sanda.popescu@umfcv.ro (S.M.P.); 2 Department of Medical Informatics and Biostatistics, University of Medicine and Pharmacy of Craiova, 200349 Craiova, Romania; 3 Department of Histology, University of Medicine and Pharmacy of Craiova, 200349 Craiova, Romania; cristina.andrei@umfcv.ro; 4 Department of Plastic and Reconstructive Surgery, University of Medicine and Pharmacy of Craiova, 200349 Craiova, Romania; razvan.mercut@umfcv.ro; 5 Department of Automotive, Transports and Industrial Engineering, Faculty of Mechanics, University of Craiova, 200585 Craiova, Romania; oana.gingu@edu.ucv.ro; 6 Department of Engineering and Management of Technological Systems, Faculty of Mechanics, University of Craiova, 200512 Craiova, Romania; gabriela.sima@edu.ucv.ro; 7 Department of Academic Ethics and Legislation, University of Medicine and Pharmacy of Craiova, 200349 Craiova, Romania; lavinia.tumbar@tcpa.ro

**Keywords:** biomaterials, xenograft, synthetic biomaterial, bone regeneration, bone graft materials, microscopic analysis, histological analysis, rat calvaria

## Abstract

(1) Background: Alveolar bone regeneration in dentistry has become important with the evolution of implantology. Biomaterials used for bone grafting are increasingly used to provide resistant bone support that is favorable for the insertion of dental implants. The aim of the study was to analyze the degree of biocompatibility and bone neoformation of two biomaterials compared to natural healing. (2) Methods: Bone defects of 3 mm diameter were created in the calvaria of 15 adult male Wistar rats. Three groups were created: group A, in which natural healing was achieved; group B, in which porcine xenograft was added; and group C, in which experimental synthetic bone based on hydroxyapatite reinforced with titanium particles was added. Samples were collected at 2 and 4 months postoperatively and analyzed microscopically and histologically. (3) Results: Data were obtained on the healing pattern of the created cavities, as well as the degree of their filling with newly formed bone tissue. Following the results obtained from the stereomicroscope analysis and histological analysis, statistically significant differences were observed between the two biomaterials regarding the time required for the transformation process of the graft particles into bone. Thus, the porcine xenograft was incorporated more quickly into the native bone, while the synthetic bone required a longer period of time. (4) Conclusions: The bone graft materials used acted as scaffolds for the newly formed bone, but each biomaterial required a different amount of time for the particles to be incorporated into the native bone.

## 1. Introduction

Over time, a wide range of techniques and biomaterials have been used to support the regeneration process of postextraction alveolar bone, with the aim of providing a resistant bone structure [1]. The main goal of researchers in the field of regenerative dentistry is to find effective techniques and biomaterials for healing bone defects so as to obtain sufficient bone volume, which is also qualitative, to be able to provide the stability needed in implant treatments [2]. The gold standard regarding biomaterials used for grafting bone defects is represented by autologous bone grafts [3,4,5,6]. A wide range of other bone grafting biomaterials are used for the formation of new bone tissue. The use of these biomaterials is based on their osteogenetic, osteoinductive, and osteoconductive properties [7]. Viable alternatives, widely used in Europe, are represented by xenografts of animal origin and alloplastic materials [4,8]. In order to be able to use them in daily dental practice, it is important to know the properties and behavior of these materials, starting with their “in vitro” and “in vivo” testing [9].

Xenografts derived from animal tissues are effective options for bone reconstruction, resistant to resorption, and present a low risk of disease transmission [10,11]. The most used xenografts come from porcine and bovine tissues due to their similarity to human bone in terms of chemical and structural composition [12]. They are commercially available in the form of particles with a porous and trabecular structure [13]. Porcine xenografts show osteoconductive properties, and the development of different formulations intends to improve bone regeneration [14].

Host tissues must be able to modulate inflammatory responses and accept the properties released by synthetic biomaterials [15]. Hydroxyapatite is a bioactive material that possesses chemical and structural properties similar to natural bone, which gives it the advantage of chemical adhesion [16,17,18]. Its osteoconductive and osteoinductive properties have been confirmed in numerous studies in the literature [19,20,21]. The hydroxyapatite-based nanocomposite shows good adhesion of primordial cells that invade the defect in the initial stage, good cell differentiation, and proliferation [22]. Due to the slow resorption rate, hydroxyapatite has deficiencies in the process of bone neoformation, but the advantage lies in the fact that it is able to maintain its structure for a longer period of time [15,23,24].

To our knowledge, there are few studies in the literature in which stereomicroscopy has been used to assess the degree of bone neoformation during the bone regeneration process. It can examine the external structure of bone fragments and graft materials to observe the changes produced during the bone regeneration process [25,26,27]. It can be useful for initial macro- and micro-morphological analysis, but for detailed investigations, additional methods such as histomorphometry should be used. Histomorphometric analysis is one of the essential tools for assessing the changes produced in bone tissue, providing data on structure, formation (modeling and remodeling), resorption, and mineralization [28,29,30]. This technique is suitable pre-clinically for studies on animal models. Regarding the choice of the type of experimental model, the rat calvaria is widely accepted for screening osteogenic, osteoconductive, and osteoinductive technologies [31,32]. Critical bone defects are those in which healing is not completed spontaneously and which require the use of graft materials that act as scaffolds to achieve reconstruction and stimulate bone neoformation [33].

The morphology, particle size, and chemical composition of a bone graft material greatly influence its resorption rate [34]. Also, porosity and pore size play an important role in the effectiveness of bone grafts in terms of mechanical strength. Porous materials mediate bone remodeling, assisting vascularization, osteointegration from adjacent bones, and the infiltration of osteoblasts and osteoclasts [35,36].

The aim of the study was to analyze the degree of biocompatibility and the mode of bone neoformation of two biomaterials, a porcine xenograft and an experimental synthetic material based on hydroxyapatite reinforced with titanium particles, compared to natural healing. The null hypothesis is that bone regeneration occurs in the same way in the three groups studied.

## 2. Materials and Methods

### 2.1. Study Design

In the present study, bone formation degree using two different bone grafting materials was analyzed and compared to spontaneous healing after creating a bone defect. The experiment was performed on Wistar rat calvaria, in which the results of the experiment were analyzed at 2 months and 4 months. The study was performed on laboratory animals within the Animal Unit of the University of Medicine and Pharmacy of Craiova, with the study protocol being approved by the Ethics Committee of the University of Medicine and Pharmacy of Craiova (Approval Number 230/28.11.2022, approval date: 28 November 2022).

### 2.2. Study Groups

In the present study, a commercially available porcine bone graft material (THE Graft™, Purgo, Seongnam-si, Republic of Korea), an experimental hydroxyapatite nanocomposite reinforced with titanium-based particles, HAT10 [37], and natural healing were studied comparatively. Fifteen adult male Wistar rats weighing 300 g (range: 250–350 g) were used for the experiment, a study lot similar to those used in previous studies [38,39,40]. The animals were randomly divided into three groups: the negative control group (Group A, *n* = 5), to which natural healing was followed; the positive control group (Group B, *n* = 5), to which the porcine xenograft was added; and the study group (Group C, *n* = 5), to which an experimental synthetic material, a hydroxyapatite nanocomposite reinforced with titanium-based particles, was added. All rats were housed in standard cages, with food and water provided ad libitum, following the recommendations and regulations of the Sanitary Veterinary and Food Safety Directorate, Dolj, Romania. At 2 and 4 months postoperatively, animals in each group were euthanized, and tissue samples were taken.

### 2.3. Biomaterials

The analysis of the obtained results was carried out by parallel evaluation using the same methodology. A commercial xenograft and an experimental synthetic alloplastic material based on hydroxyapatite reinforced with titanium-based particles were studied. The porcine xenograft used was THE Graft™ Granules 0.25–1 mm (Purgo, Seongnam-si, Republic of Korea), which had a volume of 0.25 g and a size of 0.6 cc. This granulation is indicated for the augmentation of minor bone defects. The material is available in the form of spongy granules, packaged in vials sterilized by gamma irradiation. The experimental synthetic material used is a nanocomposite based on hydroxyapatite reinforced with titanium-based particles, obtained by two-stage sintering (TSS) of a mixture of powders (a hydroxyapatite powder and a titanium hydride powder), HAT10 [37]. It can be used for bone regeneration due to its homogeneous and densified nanostructure, high stability of the hydroxyapatite component, good biocompatibility, and high wear resistance [41].

### 2.4. Surgical Procedure

**Figure 1:**
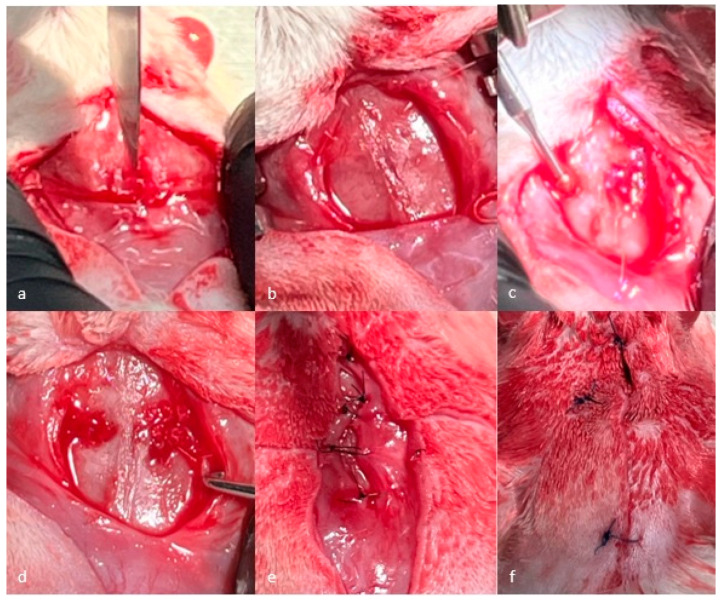
Stages of the surgical intervention.(**a**) Incision. (**b**) Flap elevation. (**c**) Creation of the defect. (**d**) Created defects—to the left of the midline is a defect created and left ungrafted; to the right of the midline is a defect created and grafted with bone granules. (**e**) Sutured periosteum. (**f**) Sutured scalp.

The surgical procedure began by performing general anesthesia of the laboratory animals, which was induced by intraperitoneal administration of Ketamine 100 mg/mL 20 I.U. (90 mg/kg) (Ketabel, Belapharm GmbH& Co. KG, Vechta, Germany) and Xylazine 2% 0.3 mL (10 mg/kg) (Xylazin Bio, Bioveta, Czech Republic), and to complete the general anesthesia, Lidocaine 20 mg/mL (4 mg/kg) (Xilina, Zentiva, Prague, Czech Republic) was used, administered subcutaneously at the level of the incision area. After scalp shaving and disinfection with Betadine 100 mg/mL (Egis Pharmaceutical, Budapest, Hungary), an incision was made in the antero-posterior direction, a flap was raised, and the bone defect was created using spherical burs with a diameter of 3 mm and mounted on the surgical handpiece under continuous irrigation with sterile saline. To prevent complete spontaneous healing of the bone, a calvarial defect of 3 mm in diameter was created, taking great care not to damage the dura mater during the surgical procedure. In order to avoid differences in the position of the bone defects, the midline was established as a reference point, with bone defects being created in the parietal bone on either side of the midline. After completion of the biomaterial addition procedure, the periosteum was sutured with 6-0 sutures (Supramid, SMI sutures, Sankt Vith, Belgium), and the scalp was sutured with 3-0 sutures. (Supramid, SMI sutures, Sankt Vith, Belgium). The incisions were covered with Betadine to avoid secondary infections (Figure 1).

After surgery, the animals were housed in well-ventilated cages with a 12 h light/dark cycle at a temperature of 25 ± 1 °C and fed with a combined pelleted food and water ad libitum. Subcutaneous analgesics with buprenorphine 0.3 mg/mL (0.05 mg/kg) (Buprenex, Hospira, Lake Forest, IL 60045, USA) were administered twice daily for 48 h.

The surgical interventions followed the previously established protocol. During the healing period, no remarkable events occurred, and no local or systemic complications were detected following the surgical intervention. The samples collected from the study groups followed a common pattern of evolution, with differences in the rate of bone neoformation, as well as the morphological elements observed at 2 and 4 months.

### 2.5. Bone Sample Preparation

**Figure 2:**
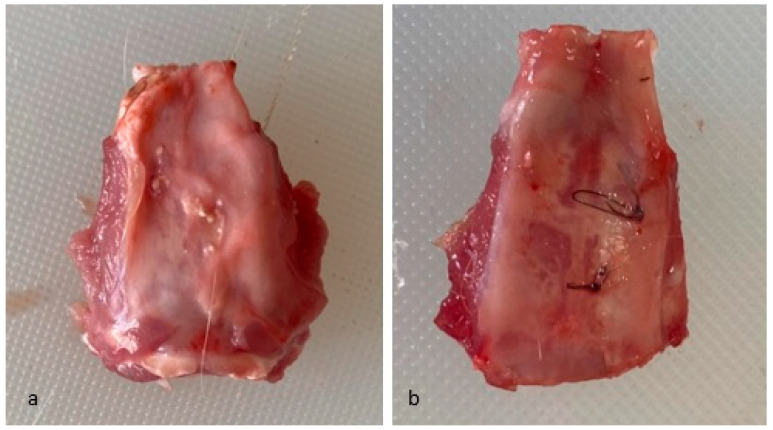
Bone samples. (**a**) Bone sample collected at 2 months. (**b**) Bone sample collected at 4 months.

The animals were randomly divided into two distinct groups; some were euthanized at 2 months and the rest at 4 months. The euthanasia of laboratory animals was performed according to current standards by anesthesia overdose. Bone samples were collected at 2 months and 4 months and prepared to include the defect along with surrounding tissues (Figure 2).

**Figure 3:**
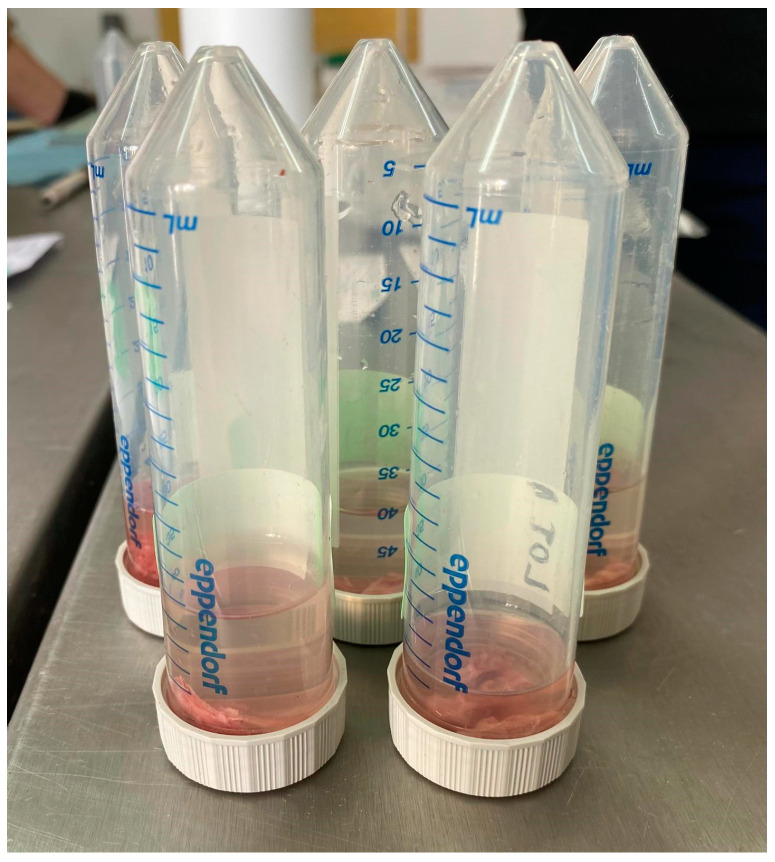
Bone samples immersed in 10% formalin.

After harvesting, the samples were washed and fixed by immersion in 10% neutral buffered formalin (Bio-Optica, Milan, Italy) for 48 h (Figure 3).

### 2.6. Microscopic Analysis

**Figure 4:**
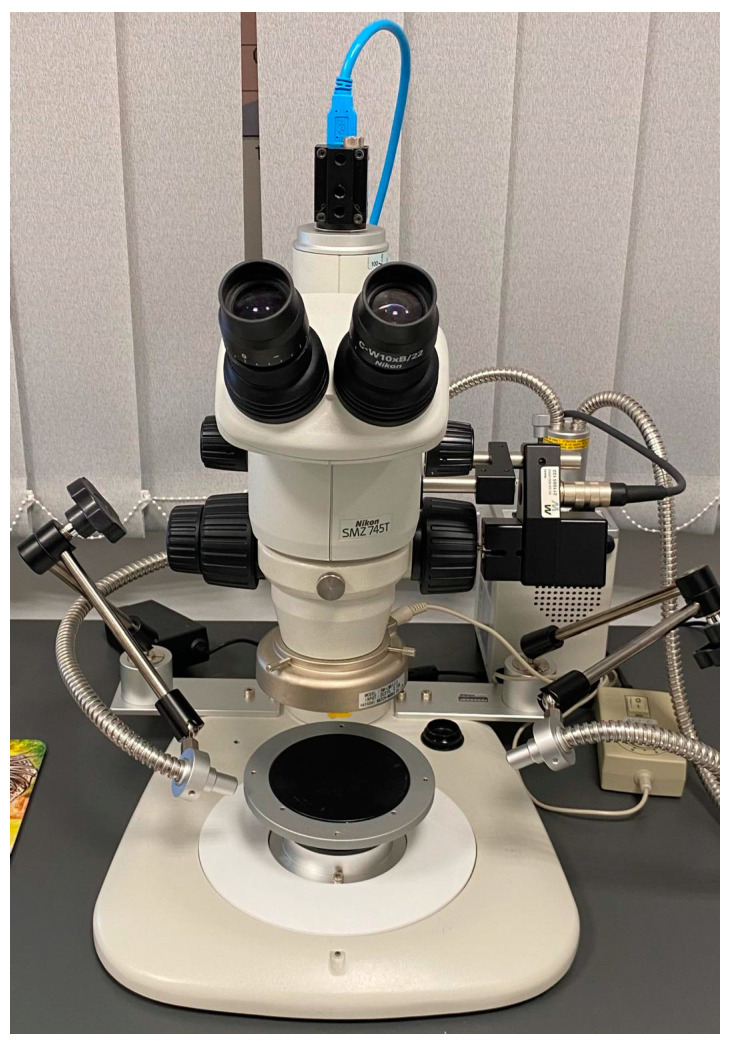
Stereomicroscope.

**Figure 5:**
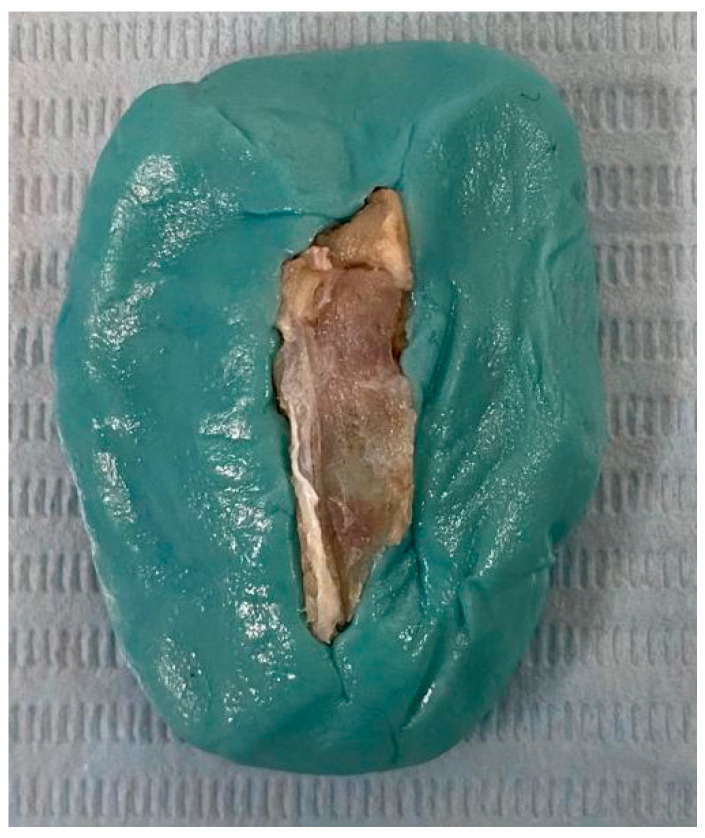
Bone samples fixed in silicone.

Before the stereomicroscopic examination, the samples were washed under running water and dried carefully. For microscopic analysis, a NIKON SMZ 745T stereomicroscope (Nikon Corporation, Tokyo, Japan) was used at a maximum magnification of 75× and a working distance of 115 mm (Figure 4). The device was used to examine the surfaces of the obtained samples, resulting in 2D images of them, stored in JPG format. To position the samples for stereomicroscopic analysis, each bone fragment was fixed in high-consistency silicone (Zetaplus L Intro KIT, Zhermack, Rome, Italy), leaving only the area of interest visible (Figure 5).

### 2.7. Analysis of Stereomicroscopic Images Using Image J

**Figure 6:**
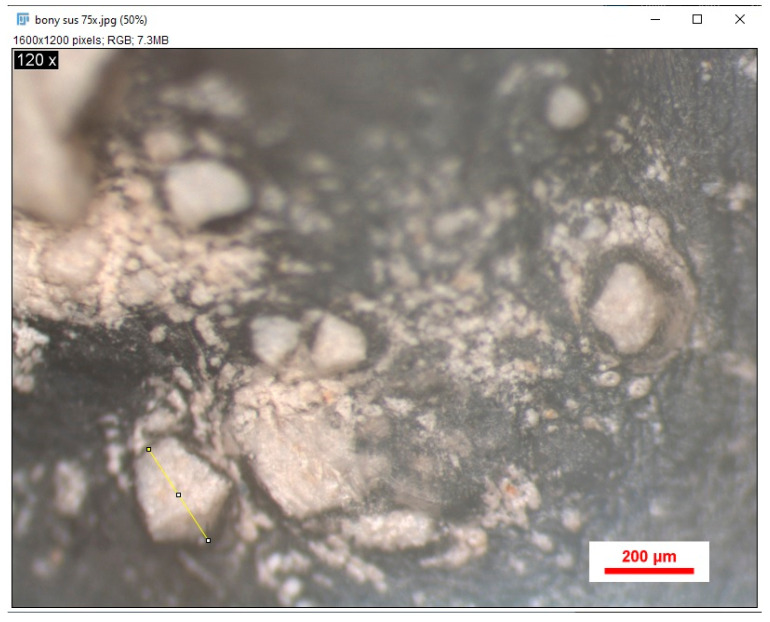
Stereomicroscopic image analyzed using Image J.

Particle size analysis of the biomaterials used was performed on the images of the samples obtained after stereomicroscopy using Image J software (version 1.54j). Initially, the scale was set to 0.2 mm, and then the residual particles observed in the images obtained for each study batch were measured (Figure 6).

### 2.8. Histologic Analysis

For histological analysis, anatomical specimens were fixed in 10% neutral buffered formalin for 48 h, washed with distilled water, and decalcified with 14% ethylenediaminetetraacetic acid (EDTA) solution at 4 °C, which was changed daily for the first 5 days. The samples were maintained for another 10 days in a 10% EDTA solution. The decalcification process was monitored by using a needle to check the consistency of the bone tissue and the progress of decalcification. The final moment of the decalcification process was established when the sample became sufficiently soft without showing any signs of mineralization. After the bone specimens became flexible, they were rinsed with phosphate-buffered saline for 30 min and high-purity distilled water for 30 min and stored in 70% ethanol at 4 °C for 2 weeks. The specimens were subsequently embedded in paraffin, sectioned with a microtome into 5 μm sections, and mounted on glass slides. Sections were examined to ensure that their thickness was consistent and to prevent any variation that could influence histological analysis.

For hematoxylin–eosin (HE) staining, the protocol involved deparaffinizing the sections in four xylene baths for 60 min each, rehydrating the sections by immersing the slides in several baths of ethyl alcohol of decreasing concentration (absolute alcohol three baths of 60 min each, 95% alcohol three baths of 120 min each, 80% alcohol one bath of 60 min) and washing in running water for 5 s. For nuclei staining, Mayer’s hematoxylin was used for 2 min; then, the samples were washed with running water for 5 s. For cytoplasm staining, eosin was used for 45 s, and then the samples were washed with running water for 5 *s* and distilled water for 5 min. The next step was dehydration using a decreasing concentration of ethyl alcohol solution for 30 min, and the samples were clarified by performing two xylene baths for 5 min. Mounting between the slide and the coverslip was performed using Entellan mounting medium (Merck & Co, Inc., Rahway, NJ, USA). Subsequently, the samples were examined using an Olympus CX 20 microscope (Olympus Corporation, Tokyo, Japan), which was attached to a camera and a computer. The obtained images were saved in JPG format.

### 2.9. Analysis of Histologic Images Using Image J

The analysis of the histological images of the obtained samples was performed using Image J software with the Bone J plugin. Data on the bone surface, the average thickness of the trabeculae, and the average diameter of the osteocytes were obtained. No volumes were calculated since the method used is based on the evaluation of two-dimensional sections. For the bone/medullary space ratio, the total area of the section was measured in µm^2^ and calculated using the following formula:

$$BS/TS=\frac{bone\;surface}{total\;surface},$$

where *BS* means bone surface, and *TS* means total surface.

The average thickness of the bone trabeculae was calculated using the following formula:

$$\frac{Ƹ\;trabeculae\;thickness}{number\;of\;measurements}$$

### 2.10. Statistical Analysis

Data obtained from measurements made with Image J were collected and analyzed using Microsoft Excel 365, version 2503 (San Francisco, CA, USA) and SPSS (Statistical Package for Social Sciences) software application, version 26 (SPSS Inc., Armonk, NY, USA). Quantitative data were expressed as “means ± standard deviation (SD)”. The data series was analyzed for normality based on the Kolmogorov–Smirnov/Shapiro–Wilk test and for variance based on Levene’s test of equality of variances. According to the obtained results, the variation of parameters and the differences between groups were analyzed based on one-way ANOVA for group comparisons (followed by Tukey post hoc analysis) and the *t*-test, with the significance threshold set at *p* < 0.05 (95% confidence level) and *p* < 0.01 (99% confidence level).

## 3. Results

### 3.1. Results of Stereomicroscopic Analysis of Samples

**Figure 7:**
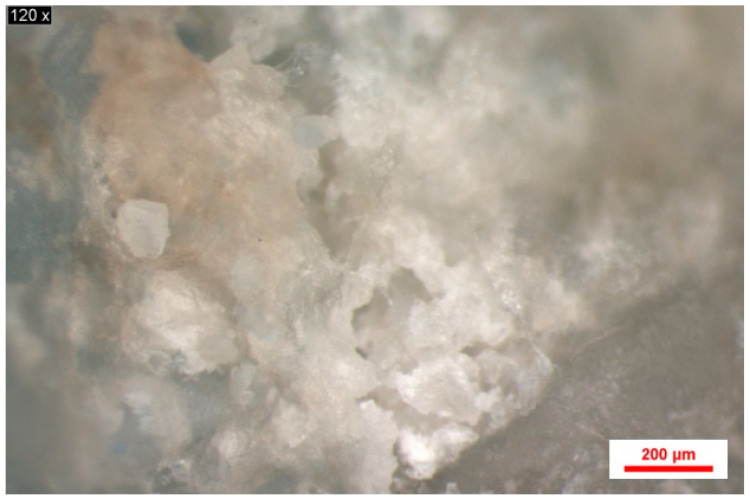
Stereomicroscope image magnified 75×.

A series of images of samples obtained from the rat calvaria, following stereomicroscopy, were analyzed using Image J software (National Institutes of Health (NIH), Maryland, MD, USA). These images show the healing stage of the bone defect, as well as traces of biomaterial particles not yet transformed into new bone tissue (Figure 7).

**Figure 8:**
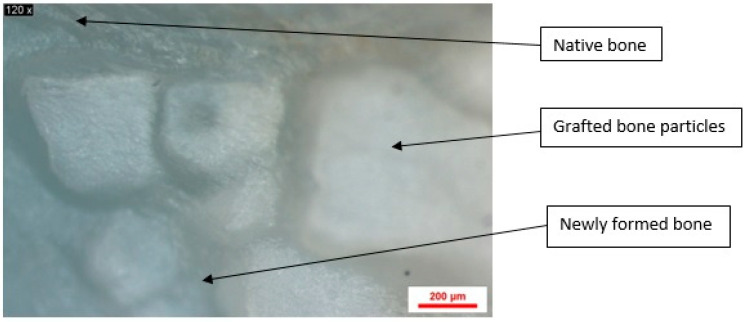
Stereomicroscope image magnified 75×. Different bone layers—native bone, grafted bone particles, newly formed bone.

For each image, a region of interest was chosen to encompass the three bone areas, namely the native bone, the grafted bone particles, and the newly formed bone (Figure 8).

**Figure 9:**
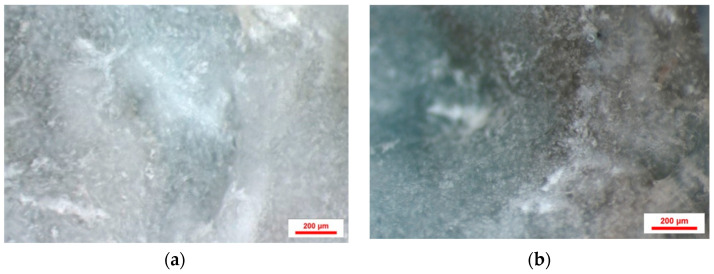

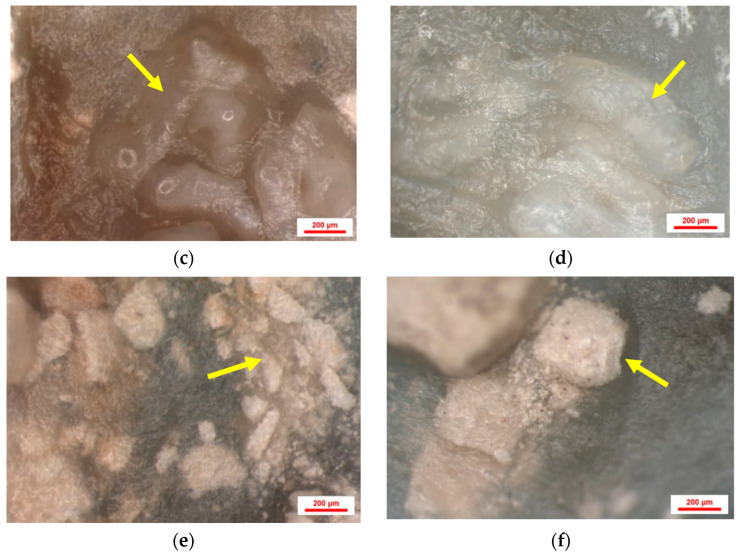
Stereomicroscope images magnified 75×. Yellow arrows mark defect areas grafted with biomaterials. (**a**) Negative control group 2 months. (**b**) Negative control group 4 months. (**c**) Positive control group (porcine xenograft) 2 months. (**d**) Positive control group (porcine xenograft) 4 months. (**e**) Study group (synthetic hydroxyapatite reinforced with titanium particles) 2 months. (**f**) Study group (synthetic hydroxyapatite reinforced with titanium particles) 4 months.

A series of images of samples collected from the three study groups from the two follow-up periods were selected to highlight the differences between the control group and the two study groups regarding the stage of healing and the amount of residual biomaterial particles (Figure 9). It was thus observed that in the negative control group, the defect was highlighted by the dark areas that showed bone tissue in the neoformation stage (Figure 9a,b). In the case of the porcine xenograft, on the stereomicroscopic image at 2 months (Figure 9c, Table 1), biomaterial particles with average dimensions of 0.416 mm were observed that were incorporated into the area of the neoformation bone tissue. In the following image (Figure 9d, Table 1), it was observed that at four months, the newly formed bone transformed into native bone, and the biomaterial particles with an average size of 0.331 mm began to have a homogeneous structure, which had a higher density than the native bone. In the image corresponding to the samples from the study group, in which synthetic biomaterial based on hydroxyapatite reinforced with titanium particles was added, at 2 months (Figure 9e, Table 1), biomaterial particles with an average size of 0.364 mm were observed, very well delimited from the newly formed bone tissue. A large part of these particles remained untransformed into bone tissue during this period of time. In the image corresponding to the samples from the study group at 4 months (Figure 9f, Table 1), it was observed that the number of biomaterial particles with an average size of 0.238 mm decreased, and areas of newly formed bone tissue appeared around them.

The comparison between the results obtained from the measurements of the biomaterial particles in the two groups and between the two follow-up periods is presented in Table 1. It was observed that the average size of the biomaterial particles decreased from two months to four months, and this transformation was statistically significant for the synthetic biomaterial particles (*p* = 0.026). The comparison of the measurements between the two groups at two months, although they showed a larger average particle size in the negative control group than in the study group, did not present statistical significance. In contrast, when comparing the particle size measurements of the two groups at four months, a statistically significant difference was observed, with the synthetic biomaterial particles having smaller sizes (*p* = 0.039). However, on stereomicroscopy images, it was observed that the degree of transformation of these particles into newly formed bone was lower for the synthetic bone based on hydroxyapatite reinforced with titanium particles compared to the porcine xenograft (Table 1).

### 3.2. Results of Histological Analysis of Samples

**Figure 10:**
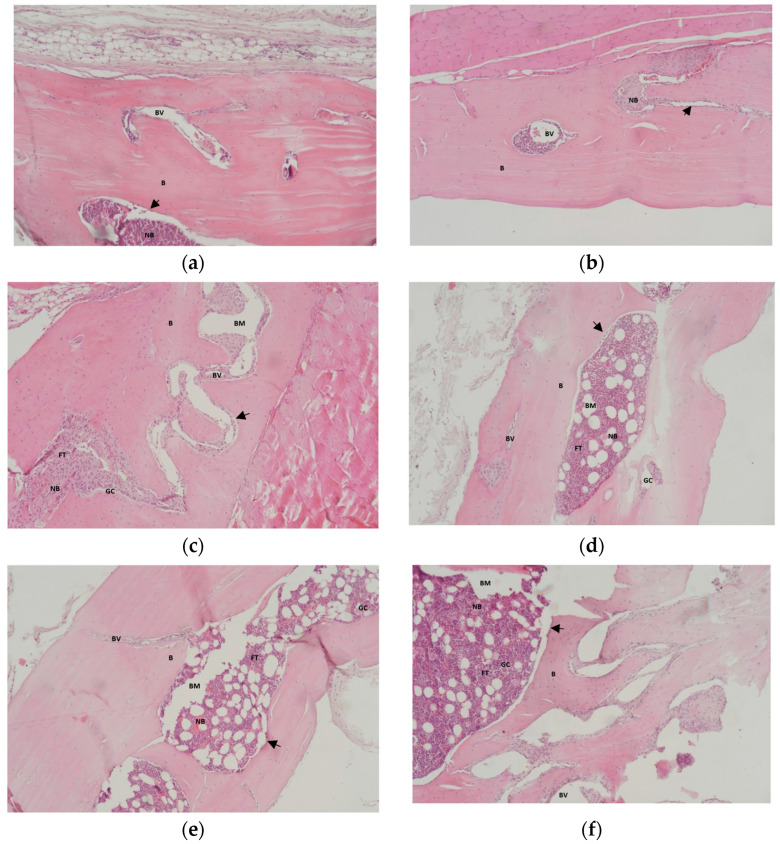
Hematoxylin–eosin 10x staining of the regions of interest of the calvarial bone defects in the following groups: negative control group (**a**,**b**), positive control group (porcine xenograft) (**c**,**d**), and study group (synthetic hydroxyapatite reinforced with titanium particles) (**e**,**f**) at 2 and 4 months. Legend of abbreviations: B—native bone, NB—newly formed bone, BM—biomaterial, BV—blood vessel, FT—fibrous tissue, GC—giant cells, arrow (↑)—indicates the row of osteoblasts that delimit the areas of newly formed bone. (**a**) Negative control group at 2 months—trabecular bone, with younger areas of bone alternating with lamellar areas. (**b**) Negative control group at 4 months—lamellar trabecular bone with hematogenous marrow and megakaryocyte giants. (**c**) Positive control group (porcine xenograft) at 2 months—trabecular bone with medullary aplasia and reactive endosteal cells. Active bone formation. (**d**) Positive control group (porcine xenograft) at 4 months—area of intramatrix remodeling, with the presence of hematogenous marrow with megakaryocytes. (**e**) Study group (synthetic hydroxyapatite reinforced with titanium particles) at 2 months—microscopic structure of trabecular lamellar bone showing intra-trabecular hematogenous bone marrow and fine areas of remodeling. (**f**) Study group (synthetic hydroxyapatite reinforced with titanium particles) at 4 months—extensive areas of confluent endo-periosteal remodeling/bone synthesis.

In the images from the negative control group, large areas of native bone were observed at both 2 and 4 months, during which osteocytic lacunae were highlighted. In the areas of bone defect, blood vessels accompanied by blood cells were observed. Near the areas of newly formed bone, laminae of osteoblasts were observed (Figure 10a,b). In the images corresponding to the positive control group, areas of native bone with filled osteocytic lacunae were observed, with newly formed blood vessels surrounding the biomaterial particles. In their vicinity, osteoblast laminae and areas of newly formed bone were observed. The dimensions of the biomaterial particles were larger in the case of images from 2 months, decreased significantly, and became rounded in images from 4 months. These particles are surrounded by areas of newly formed bone tissue. The areas of newly formed bone tissue were larger at 2 months and decreased at 4 months, transforming into native bone (Figure 10c,d).

Regarding bone surface, there were statistically significant differences in intra-group comparisons between the two follow-up periods for the positive control group (*p* = 0.047) and study group (*p* = 0.006) but not for the negative control group (Table 2). The results of comparisons regarding the maximum diameter of trabeculae and osteocytic lacunae between the two follow-up periods for all three groups were not statistically significant (Table 2). Areas of native bone tissue, blood vessels, and newly formed bone tissue were also observed in the images corresponding to the study group. In contrast to the positive control group, the number of blood vessels in the 2-month images was reduced, and it increased in the 4-month images. The sizes of the biomaterial particles, with diameters smaller than those of the porcine xenograft, were observed to decrease in the 2-month images compared to the 4-month images. The biomaterial particles were surrounded by newly formed bone tissue and rows of osteoblasts. The rate of transformation of the biomaterial particles was slower than in the porcine-derived xenograft (Figure 10e,f).

In addition, one-way ANOVA tests were run to identify whether the three parameters differed for the three bone types for each studied period. For all three groups, the analysis of all measured values revealed that there were no outliers; data was normally distributed for the type of bone, according to the Shapiro–Wilk test (*p* > 0.05), and there was homogeneity of variances, according to the Levene’s test of homogeneity of variances (*p* > 0.05. At two months, there were statistically significant differences in comparisons made between the three groups only for the bone surface area (*p* = 0.019) but not for the maximum diameter of the trabeculae and the maximum diameter of the osteocytes (Table 2). Tukey post hoc analysis revealed that the multiple group comparisons were statically significant between the study group and both the positive control group (*p* < 0.0005) and negative control group (*p* = 0.008) but not between other group combinations. At four months, there were statistically significant differences in comparisons made between the three groups for all three parameters (Table 2). Post hoc analysis revealed statistically significant differences for bone surface again between the study group and both the positive control group and negative control group (*p* < 0.0005). The mean thickness of the trabeculae was statistically significantly different between the positive control group and the negative control group (*p* = 0.003) but not between other group combinations, while the mean diameter of the osteocytes was statistically significantly different between the study group and the negative control group (*p* = 0.041) but not between other group combinations.

## 4. Discussion

The study used adult male Wistar rats. Adult males were chosen because young animals have a higher potential for the spontaneous repair of created bone defects, and females can influence the results through hormonal interference [42]. The experimental model used was the rat calvaria, which is considered the gold standard from an embryological point of view in the evaluation of changes produced by bone grafting materials in dentistry [14]. The healing interval chosen was between 2 and 4 months in order to capture the process of bone formation, as well as the moment of biomaterial resorption [42]. Following the stereomicroscopy study in which the morphometry of biomaterial particles was analyzed at 2 and 4 months, their persistence was observed, with a decrease in size over time. The porcine xenograft showed a higher incorporation into the newly formed bone compared to the synthetic material based on hydroxyapatite reinforced with titanium particles, which, although its particles shrank more, was less incorporated into the newly formed bone.

The ideal grafting biomaterial for bone defect augmentation should be considered the one that has the properties closest to those of natural bone so as to ensure the growth and differentiation of bone tissue, as well as its vascularization [43]. Regardless of the type of biomaterial used, when evaluating the bone regeneration procedure, in addition to the characteristics of the particles, their compaction and localization are also important, with these aspects being closely related to the size and shape of the particles [44]. The particle size of the biomaterials is especially important for the waiting time between resorption and replacement with new bone [2]. The presentation form in terms of bone particles lacks bonding strength, which leads to poor handling and can ultimately lead to displacement or loss of the material during the filling of the bone defect [45,46]. Recently, novel approaches, such as the 3D printing of scaffolds, have been studied in the literature. These have become increasingly relevant in bone tissue regeneration due to the ability to customize the architecture and composition of the materials according to the patient’s needs [47,48]. In this context, composite materials, such as hydroxyapatite reinforced with titanium particles, show great potential for integration into such technologies.

In the study conducted by Takauti et al., the researchers compared animal xenografts with synthetic biomaterials in critical defects made on rabbit calvaria. They observed that synthetic biomaterials showed higher rates of new bone formation compared to xenografts [49]. In the study by Falacho et al., porcine xenografts tested showed favorable biocompatibility, appearing to undergo extensive processes of resorption, demineralization, and particle disintegration that may lead to the replacement of the biomaterial with new bone formation [14].

The histological images of the samples confirmed the findings made following the morphometric analysis of the stereomicroscopy images. Thus, in the case of the porcine xenograft, a larger size of the newly formed bone areas was observed at 2 months, while in the images at 4 months, the newly formed bone was present, completely surrounding the shrunken biomaterial particles, and the newly formed bone at 2 months transformed into native bone. In the case of the histological images from the study group, the biomaterial particles at 4 months had shrunk much more compared to those at 2 months, being surrounded by newly formed bone tissue over large areas. The histological image from the study group at 4 months is not that much different from that at 2 months, which shows that the progress in the incorporation of these biomaterials is slower than in the case of porcine xenografts. In the literature, numerous studies have stated that histological analysis is an important method for evaluating hard bone tissues [50]. In studies involving both xenografts and synthetic materials, dense polymorphonuclear cell infiltration and acute inflammation were frequently found. Slowly resorbable materials, such as hydroxyapatite, can cause chronic inflammation and can even be encapsulated by fibrous tissue [51].

The study by Draghici et al. showed a common pattern of evolution of biomaterials used for bone grafting, with differences in the rate of osteoformation and different morphological elements between the two time points. In the case of the biomaterials used in their study, ossification started from the center of the created cavity, away from the edges of the bone defect, with osteon-like structures present. In addition, the experimental material showed residual particles and a more intense initial inflammatory reaction, which determined the appearance of a dense-looking and less uniform bone repair tissue [38].

In the research conducted by Bielenstein et al., which studied the regenerative capacity and immune response following the grafting of two biomaterials into defects created in rat calvaria, namely a xenograft of bovine origin and a synthetic material, the results showed that the grafting of the synthetic material induced a greater pro-inflammatory response of the tissue, while the bovine xenograft induced a greater anti-inflammatory reaction, and comparable amounts of bone regeneration were observed in both study groups [52].

In a study by Bae et al., porcine xenografts were compared with bovine xenografts being used in defects created in rat calvaria. Their results showed that human mesenchymal stem cells differentiate osteogenically without toxicity, similar to the results of their study [53]. In the study by Naini et al. [10], in which two xenografts added to bone defects created in rabbit calvaria were compared, all study groups showed mild inflammation, suggesting high biocompatibility and minimal immune response to the grafted materials, which is consistent with other previous studies [54,55].

An important aspect to consider during sample analysis is the residual particles of the biomaterials. These prevent complete resorption of the biomaterial before the new bone formation occurs, but it is important that they do not remain incorporated longer than necessary, affecting the replacement with new bone formation [56]. Numerous studies have been proposed in the literature related to the reconstruction of large bone defects, which are difficult to manage due to the lack of vascularization [57], but in daily practice, smaller bone defects are much more common [38], with their optimal healing being of particular importance for the long-term success of the implant treatment [58,59]. Porcine xenografts are still undergoing research to evaluate their potential as bone substitutes since they originate from an animal species with a genotype close to that of humans. The Ca/P ratio of this type of xenograft is similar to the Ca/P ratio of human bone [60], and the bone strength is approximately 200 ± 300 MPa, which is close to the 50 ± 389 MPa strength of human bone [61]. Various studies in the literature have shown that these materials provide an effective osteoconductive matrix [62,63], thus confirming their biocompatibility [64]. The porcine xenograft used in this study is a porous mineral matrix, which is produced by removing all organic components from porcine bone. Due to its natural structure, it compares to the physical and chemical aspects of the mineralized matrix of human bone [65]. In the study conducted by Kasuya et al., bone defects were grafted with bovine xenograft. They observed that the grafted biomaterial acted as a scaffold for the repair of bone defects [66].

Compared to porcine grafts, bovine grafts have a slower resorption rate and greater structural durability, making them suitable for areas with long-term mechanical strength needs. However, the use of bovine xenografts may induce a more pronounced inflammatory reaction and the formation of a fibrous capsule around the graft, risks that are reduced with porcine grafts [38]. Also, synthetic hydroxyapatite, although highly biocompatible due to its chemical composition similar to that of human bone, has a slower degradation rate, which may lead to insufficient new bone formation in the absence of osteoinductive proteins. In addition, previous studies have shown that while porcine grafts favor rapid and efficient integration, they resorb more quickly than synthetic hydroxyapatite, which may represent an advantage in certain applications but also a limitation in others, depending on the clinical goal pursued [14]. Long-term risks associated with the biocompatibility of graft materials, such as chronic inflammation and fibrous capsule formation, should be considered when considering the use of porcine xenografts and titanium-reinforced synthetic hydroxyapatite. Previous studies have shown that these biomaterials can induce a long-lasting inflammatory reaction if they are not fully integrated into the bone, which can lead to the formation of a fibrous capsule around the graft. The formation of foreign body giant cells is also an indicator of a persistent immune reaction to xenogeneic materials. In the case of porcine xenografts, the risks related to immune reactions are lower than in the case of xenografts of bovine origin due to their biological structure, which is closer to human bone, but they are not completely eliminated. Regarding synthetic hydroxyapatite, long-term stability is also an important factor to consider. According to recent studies, hydroxyapatite can cause a moderate inflammatory reaction, which is usually self-limited, but there is a risk that, in the long term, a fibrotic reaction may form at the material–bone interface. In this context, histological analyses should include the assessment of the presence of foreign body giant cells and fibrous tissue around the graft to adequately assess the long-term biocompatibility of these materials [8].

The results of our study showed a slower degradation rate and, implicitly, a reduced osteointegration of synthetic hydroxyapatite reinforced with titanium compared to a porcine xenograft. These differences in the osteointegration mechanism could be influenced by several factors, such as the porosity of the materials, the surface chemistry, and the interactions of titanium particles with body cells. Synthetic hydroxyapatite, although it has a chemical composition similar to human bone, has a lower porosity than porcine xenografts, which may lead to a slower osteointegration, given that bone cells integrate more quickly into porous structures that favor cell migration and vascularization. Regarding titanium-reinforced hydroxyapatite, the interactions between titanium particles and bone tissue may influence the biological response. Although titanium is considered a bioinert material, titanium particles can induce local inflammatory responses, especially by modulating the activity of macrophages and polarizing them towards a pro-inflammatory phenotype. Previous studies have shown that titanium particles can affect how immunological cells respond to implanted materials, which may contribute to a more persistent inflammatory reaction and, implicitly, to poorer osseointegration [67,68].

Following the results of our study, both from the images obtained from the analysis of the samples using the stereomicroscope and from the images obtained from the histological analysis, we observed that there is a clear difference in the behavior of the bone graft particles regarding their incorporation into the bone. Thus, the porcine xenograft was incorporated more quickly and generated the appearance of larger areas of newly formed bone, which, in the histological images at 4 months, already had the structure of native bone. The synthetic material based on hydroxyapatite reinforced with titanium particles showed a different behavior. Thus, although at 4 months, this biomaterial presented particles smaller in size than the porcine xenograft, they were not incorporated as much into the native bone, and the process of bone neoformation required a longer time.

Although hydroxyapatite-based biomaterials are biocompatible and have been developed as bone substitutes and introduced into clinical use, there is currently no such ideal bone substitute [66]. Hydroxyapatite is a relatively chemically stable substance that dissolves slowly and remains for a long time in the area of the bone defect where it is grafted [69]. The grafting material based on hydroxyapatite reinforced with titanium particles shows a slow resorption rate in the biologically active environment, which, according to some authors, may represent a higher susceptibility to infections [70,71]. In our study, we also encountered residual particles of this material in all samples. However, no signs of local infection were identified. In the study by Susin et al., in which hydroxyapatite-based biomaterials were analyzed, it was found that none of the biomaterials used supported bone formation and maturation beyond the native regenerative potential of the rodent model used, indicating their limitations for regenerative procedures. They stated that the biocompatibility and dimensional stability of the biomaterial might suggest their potential utility as long-term defect fillings [8]. In the study conducted by Araujo et al., the effect of using autologous and xenograft micrografts on critical size defects created in the calvaria of rats was investigated. Histomorphometric and histological analysis showed that the group that received autologous micrografts combined with xenograft and the group that received autologous bone graft resulted in greater bone formation at both time points compared to the use of xenograft alone and blood clot [72].

The study results are promising, but translating these biomaterials into clinical practice involves a number of challenges. Regulatory approval processes for the use of xenografts or synthetic biomaterials are often complex and require a clear demonstration of biocompatibility and safety in use. Also, the cost of production and economic feasibility of materials such as titanium-reinforced hydroxyapatite need to be analyzed in the context of large-scale application in human patients [73]. Another important aspect is the stability of the graft and the potential risk of displacement, especially in anatomical areas subject to mechanical loads, in contrast to the calvarial model used in the present study.

Although the materials analyzed in this study are in granular form, their clinical application in the context of post-extraction socket regeneration is much more effective when combined with PRF (platelet-rich fibrin) [74]. The mixture of PRF and graft allows for obtaining a product known as sticky bone, which, in addition to improving the maneuverability of the additional material, also offers a favorable cost-effectiveness ratio since a smaller amount of material is used [75]. In addition, biocompatibility is significantly increased due to the presence of growth factors in PRF, which favor healing and osteogenesis [74]. These aspects support the relevance of the preclinical results obtained and may contribute to the development of effective clinical applications in bone regeneration.

Limitations of the present study lie in the fact that the samples were collected from different animals, and individual variations may interfere with the healing process. Biological variability between animals can influence their response to applied treatments and the interpretation of results. Although our study used exclusively adult male Wistar rats to minimize hormonal interference and to avoid the greater spontaneous influence of bone defect repair in young animals, this represents a limitation in generalizing the results to female subjects. Hormonal differences, such as the effects of estrogen on bone metabolism, may significantly influence the response to applied treatments, and future studies should explore sex-specific responses to understand the mechanisms of bone regeneration more fully.

## 5. Conclusions

The results showed that the bone graft materials acted as scaffolds for the newly formed bone, but it took a long time for full repair. These results, obtained for the first time on an animal model from the comparison between a porcine xenograft and a synthetic hydroxyapatite reinforced with titanium particles, show that the porcine xenograft is incorporated more rapidly and more into the bone, while the synthetic biomaterial, although it reduces its particle size, acts as a scaffold, transforming slowly and incompletely into bone.

## Figures and Tables

**Table 1:** Measurements of biomaterial particle sizes on stereomicroscopy images between the two groups in the two follow-up periods.

Sample	Measurements				P (2M–4M)
	2 Months		4 Months		
	Mean	Standard Deviation	Mean	Standard Deviation	
Positive Control Group (Porcine Xenograft)	0.416	0.063	0.331	0.037	0.057 *
Study Group (Synthetic Hydroxyapatite Reinforced with Titanium Particles)	0.364	0.060	0.238	0.042	0.026 *^,#^
	0.288 **		0.039 **^,#^		

* comparative analysis within the group at 2 months and 4 months, *t*-test. ** comparative analysis between the two materials at 2 months and 4 months, *t*-test. ^#^ statistically significant, *p* < 0.05.

**Table 2:** Measurements of histological parameters between the two groups in the two follow-up periods.

Sample	Bone Surface			Mean Thickness of the Trabeculae			Mean Diameter of the Osteocytes		
	2 Months	4 Months	*p*	2 Months	4 Months	*p*	2 Months	4 Months	*p*
Negative Control Group	102.436 ± 125.012	122.037 ± 127.383	0.221 *	8.63 ± 46.388	18.716 ± 92.024	0.120 *	15.502 ± 69.553	17.606 ± 66.883	0.472 *
Positive Control Group (Porcine Xenograft)	109.494 ± 126.222	112.129 ± 126.570	0.047 *^,#^	6.910 ± 44.401	6.674 ± 36.082	0.763 *	14.376 ± 10.317	13.917 ± 9.368	0.387 *
Study Group (Synthetic Hydroxyapatite Reinforced with Titanium Particles)	45.372 ± 97.372	77.642 ± 117.348	0.006 *^,##^	12.143 ± 38.125	10.582 ± 38.597	0.255 *	13.895 ± 13.520	12.663 ± 8.386	0.059 *
	0.019 **^,#^	0.037 **^,#^		0.128 **^,#^	0.024 **^,#^		0.685 **	0.048 **^,#^	

* comparative analysis within the group at 2 months and 4 months, *t*-test. ** comparative analysis between the three materials at 2 months and 4 months, one-way ANOVA. ^#^ statistically significant, *p* < 0.05. ^##^ statistically significant, *p* < 0.01.

## Data Availability

The original contributions presented in the study are included in the article. Further inquiries can be directed to the corresponding author.

## Abbreviations

The following abbreviations are used in this manuscript:

TSS	two-stage sintering
NIH	National Institutes of Health
EDTA	ethylenediaminetetraacetic acid
HE	hematoxylin–eosin
BS	bone surface
TS	total surface
SD	standard deviation
B	native bone
NB	newly formed bone
BM	biomaterial
BV	blood vessel
